# Migraine in women: the role of hormones and their impact on vascular diseases

**DOI:** 10.1007/s10194-012-0424-y

**Published:** 2012-02-26

**Authors:** Simona Sacco, Silvia Ricci, Diana Degan, Antonio Carolei

**Affiliations:** Department of Neurology and Regional Referral Center for Headache Disorders, University of L’Aquila, Piazzale Salvatore Tommasi, 1, 67100 L’Aquila, Italy

**Keywords:** Migraine, Hormones, Contraceptive, Pregnancy, Menopause

## Abstract

Migraine is a predominantly female disorder. Menarche, menstruation, pregnancy, and menopause, and also the use of hormonal contraceptives and hormone replacement treatment may influence migraine occurrence. Migraine usually starts after menarche, occurs more frequently in the days just before or during menstruation, and ameliorates during pregnancy and menopause. Those variations are mediated by fluctuation of estrogen levels through their influence on cellular excitability or cerebral vasculature. Moreover, administration of exogenous hormones may cause worsening of migraine as may expose migrainous women to an increased risk of vascular disease. In fact, migraine with aura represents a risk factor for stroke, cardiac disease, and vascular mortality. Studies have shown that administration of combined oral contraceptives to migraineurs may further increase the risk for ischemic stroke. Consequently, in women suffering from migraine with aura caution should be deserved when prescribing combined oral contraceptives.

**Electronic supplementary material:**

The online version of this article (doi:10.1007/s10194-012-0424-y) contains supplementary material, which is available to authorized users.

## Introduction

Migraine is a predominantly female disorder. Women, compared with men, have a 1-year migraine prevalence nearly threefold higher (17 vs. 6%) and lifetime incidence more than twofold higher (43 vs. 18%) [1, 2]. Moreover, menarche, menstruation, pregnancy, and menopause as use of oral contraceptives and of hormone replacement treatment (HRT) may influence migraine occurrence. Until puberty, migraine affects both sexes equally [3]. After the menarche there is an increasing prevalence of migraine in women [4, 5]. The mechanism for the gender difference in migraine is not clear even if endogenous sex steroids are considered to play a relevant role.

## Migraine during women’s life

**Fig. 1:**
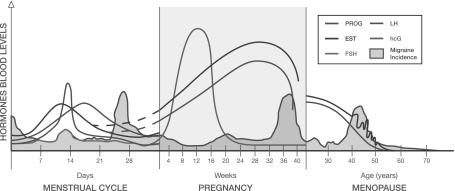
Hormonal changes and incidence of migraine without aura in women

The woman’s reproductive cycle is regulated by the hypothalamic-hypophyseal-ovarian axis through the release of estrogen and progesterone. Variations in the levels of these hormones and of their feedback control regulate the menstrual cycle, pregnancy, puerperium, and menopause (Fig. 1).

A normal menstrual cycle lasts about 28 days and consists of two phases: the follicular or the proliferative phase and the luteal or ovulatory phase. The first day of menstruation is considered the start of follicular phase and bleeding occurs after estrogen and progesterone levels decrease at the end of the previous cycle. At this time, the pituitary follicular stimulating hormone (FSH) level increases slightly, stimulating the development of several ovarian follicles. Each follicle contains an oocyte; only one follicle proceeds through ovulation producing increased levels of estrogens, which result in a drop of the FSH production, preventing the additional development of follicles, and in the stimulation of the hypophysis to release the luteinizing hormone (LH). Progesterone remains low during the follicular phase except for a small rise just prior to ovulation. At the time of ovulation, a mature follicle ruptures in response to a surge of LH, releasing a mature oocyte. The luteal phase starts just after ovulation and during this phase the follicle, denominated corpus luteum*,* secretes progesterone and estrogen, which stimulate the endometrium to prepare a thick layer of blood vessels for possible fertilization. If no pregnancy occurs, the corpus luteum persists for about 14 days and then degenerates with a fall in blood estrogen and progesterone levels and a shedding of the top layers of endometrium for the beginning of a new menstrual cycle.

When pregnancy occurs, the trophoblast releases the human chorionic gonadotropin (hCG) which allows the corpus luteum to continue to produce estrogen and progesterone until the formation of the placenta. The placenta, from that point on, produces the majority of estrogen and progesterone necessary for the pregnancy. Serum levels of estradiol and progesterone begin to rise in the mother during the 6th to 8th week of pregnancy and continue to gradually increase to their highest levels during the third trimester; serum estradiol levels during the third trimester of pregnancy are 30–40 times higher and progesterone levels are 20 times higher than their peak levels during natural menstrual cycles. The hormonal levels drop sharply during the puerperium that is defined as the time from delivery of the placenta through the first few weeks after the delivery (usually 6 weeks) and represents the phase in which the woman’s body returns back to prepregnancy condition.

The transition from the reproductive to the non-reproductive phase occurs over a period of years and is the result of a reduction in female hormonal production by the ovaries. Although the perimenopausal period is characterized by considerable fluctuations of estrogen and progesterone levels, higher than during the normal phases of menstrual cycle in the fertile period, the menopause is characterized by hormonal stability due to decline of estrogen and progesterone production by the ovaries. The average age of menopause is 51 years, within an age range of 40–60 years [6].

### Menstrual cycle

Throughout the reproductive years, menstruation is one of the most significant events related to the occurrence of migraine attacks [7, 8] (Fig. 1). Compared with all other phases of the menstrual cycle, incidence of migraine without aura (MwA) is greatest during a 5-day window that starts 2 days before the onset of menstruation and continues through the first 3 days of menstruation [9–12]. The International Classification of Headache Disorders, II edition (ICHD-II) identifies in the Appendix: (a) menstrually related migraine, which is MwA that regularly occurs on or between days −2 to +3 of menstruation, with additional attacks of migraine with aura (MA) or MwA at other times of the cycle; (b) pure menstrual migraine (MM), which is MwA that occurs only on or between days −2 to +3, with no attacks at any other time of the cycle [13] (Table 1). Pure MM is uncommon with respect to menstrually related migraine. Fewer than 10–20% of women report migraine exclusively with menstruation and at no other time of the month [9, 14–17]. Generally, the term MM includes both types of those attacks. MM attacks occur almost invariably without aura even in MA patients [7, 18, 19]. According to the majority of the available studies, compared with migraine at other times of the cycle, menstrual attacks last longer, are more severe, more likely to relapse, less responsive to treatment, and associated with greater disability [11, 12, 18, 20–24]. According to some studies, MM attacks are accompanied by nausea and vomiting more than non-menstrual attacks [18, 19] although this finding is not unanimously shared by all the studies [12, 22, 24, 25]. Correlations have been identified between premenstrual syndrome and MM [26, 27]. The association of migraine with ovulation is controversial but generally not supported despite some women may report attacks during this phase [19]. While MwA is clearly associated with menstruation, MA is generally unrelated to them [4]. Even in patients with aura accompanying their migraine attacks during the remaining of the month, the MM attacks are without aura [10, 12].

**Table 1:** Diagnostic criteria according to the International Classification of Headache Disorders, II edition, for pure menstrual migraine without aura and menstrually related migraine without aura

*A1.1.1 Pure menstrual migraine without aura*
Diagnostic criteria
A. Attacks, in a menstruating woman, fulfilling criteria for 1.1. Migraine without aura
B. Attacks occur exclusively on day 1 ± 2 (i.e., days −2 to +3)^a^ of menstruation^b^ in at least two out of three menstrual cycles at no other times of the cycle
*A1.1.2 Menstrually* *related migraine without aura*
Diagnostic criteria
A. Attacks, in a menstruating woman, fulfilling criteria for 1.1. Migraine without aura
B. Attacks occur on day 1 ± 2 (i.e., days −2 to +3)^a^ of menstruation^b^ in at least two out of three menstrual cycles and additionally at other times of the cycle

^a^The first day of menstruation is day 1 and the preceding day is −1; there is no day 0

^b^For the purposes of this classification, menstruation is considered to be endometrial bleeding resulting from either normal menstrual cycle or from the withdrawal of exogenous progestogens, as in the case of combined oral contraceptives and cyclical hormone replacement therapy

### Pregnancy and puerperium

Most epidemiological studies have demonstrated that the majority of women suffering from migraine note remarkable and increasing improvement of their attacks during pregnancy, from the first to the third trimester [7, 15, 28–30] (Fig. 1). Improvement is more likely in women with a history of MM [7, 30, 31]. If migraine does not improve by the end of the first trimester, it is likely to continue throughout the pregnancy [32]. In fact, a small number of pregnant women experience a worsening of their migraine [7, 15], while a few others may even develop de novo migraine symptoms [33]. The worsening usually occurs during the first trimester [34] and involves women suffering from MA rather than from MwA; also de novo migraines during pregnancy mostly consist of MA [14, 35].

Nearly all women report the return of migraine attacks after delivery [15, 32]. Factors accelerating the return of migraine attacks in the postpartum include bottle-feeding and age of 30 years or less [30].

Women suffering from migraine are also at higher risk of developing gestational hypertension, preeclampsia, or vascular complications related to pregnancy including ischemic stroke and other vascular events in the peripartum period [36, 37]. The risk is particularly evident in those women not showing remission or amelioration of migraine attacks. Even neonatal outcomes may be affected by the persistence of migraine [38, 39]. For all the above reported reasons, migraine should be considered a potential risk factor in obstetric care.

### Menopause

During the transition to menopause some women may experience a worsening of the migraine attacks [40], but usually, postmenopause is also associated with respite [41] (Fig. 1). The type of menopause has a substantial effect on migraine: natural menopause is associated with a lower prevalence of migraine compared to surgical menopause [15, 40, 42]. The longer is the time interval from menopause onset, the greatest is the association with improvement [41, 43]. Whether migraine is associated with other menopause symptoms is unclear since data from available studies are conflicting [43, 44]. In contrast to the effects of menopause on MwA, prevalence of MA does not improve with menopause [43].

### Contraceptives

Several forms of hormonal contraceptives are available: combination oral contraceptives (COCs), progestin-only contraceptives, 91-day combination oral contraceptives, combination patch contraceptives, and contraceptive vaginal rings. Start of hormonal contraceptives may have different effects on migraine (Table 2). The best-studied contraceptives in relation to migraine are COCs. There is a well-documented association between COCs and migraine [45]. They may induce a de novo migraine in women without a previous history of the disease, worsen a previous existing migraine, or change the pattern of a previous existing migraine (from MwA toward MA); in some cases no changes occur with COCs use [14, 15, 46]. Migraine worsening in frequency or severity has been reported in 18–50% of cases, migraine improvement has been reported in 3–35%, and no change in 39–65% of cases [47]. In COCs users in which migraine persists, attacks are more likely to occur during the pill-free week [47–51]. The addition of supplemental estradiol during the perimenstrual period, continuing the administration of hormonally active pills, or extended transdermal application is associated with an overall reduction in migraine severity and frequency [49, 51–53]. Patients suffering from pure MM are particularly sensible to COCs use and reduction of the pill-free period is associated with reduced migraine burden [54]. The use of COCs worsens to a greater extent in MA than MwA [7]. Moreover, patients may develop aura symptoms for the first time in association with the initiation of therapy [55]. The use of progesterone-only contraceptives may reduce the number of attacks in MA and may ameliorate aura symptoms in women in whom MA onset was related to previous COCs treatment [56].

**Table 2:** Diagnostic criteria according to the International Classification of Headache Disorders, II edition, for exogenous hormone-induced headache and estrogen-withdrawal headache

*8.3.1 Exogenous hormone*-*induced headache*
Diagnostic criteria
A. Headache or migraine fulfilling criteria C and D
B. Regular use of exogenous hormones
C. Headache or migraine develops or markedly worsens within 3 months commencing exogenous hormones
D. Headache or migraine resolves or reverts to its previous pattern within 3 months after total discontinuation of exogenous hormones
Comments: regular use of exogenous hormones, typically for contraception or hormone replacement therapy, can be associated with increase in frequency or new development of headache or migraine. When a woman experiences headache or migraine associated with exogenous estrogen-withdrawal, both codes 8.3.1 exogenous hormone-induced headache and 8.4.3 estrogen withdrawal headache should be used
*8.4.3 Estrogen*-*withdrawal headache*
Diagnostic criteria
A. Headache or migraine fulfilling criteria C and D
B. Daily use of exogenous estrogen for ≥3 weeks, which is interrupted
C. Headache or migraine develops within 5 days after last use of estrogen
D. Headache or migraine resolves within 3 days
Comments: estrogen-withdrawal following cessation of a course of exogenous estrogens (such as during the pill-free interval of combined oral contraceptives or following a course of replacement or supplementary estrogen) can induce headache and/or migraine

### Hormone replacement treatment

HRT has a variable effect on migraine (Table 2). Most women with headache report improvement or complete remission of their headache, associated with HRT use; a minority of women report no change or worsening of the headache [46]. Specifically, HRT is associated with an increased risk of migraine headache [57]. There are no significant differences in the risk of migraine headache in users of cyclic versus continuous doses of estrogens and no dose–response relationship has been reported [57]. HRT may trigger migraine attacks in women without a past history of migraine or may also cause the reappearance of migraine in those women who had the disorder before menopause [57, 58]. Worsening of migraine in menopause may be a factor in predicting worsening of migraine with HRT [59]. It is worth mentioning that in some cases HRT is recommended for perimenopausal women with migraine [60, 61]. Further, studies suggest that non-oral routes of delivery of estrogen are more likely to improve migraine than oral estrogens [62] probably because of lower fluctuations in estrogens levels.

HRT may have adverse effects on MA. It may worsen a preexisting MA or it may induce MA in women without history of the disease [63]. In those cases, withdrawal of estrogens and additional migraine prophylaxis lead to improvement or complete cessation of migraine [63]. Other studies reported also that MA may resolve with either a reduction in estrogen dose or change in route of delivery of estrogen (switch from oral to transdermal administration) [62].

## Mechanisms linking sex hormones and migraine

The observation that migraine is predominantly a female disorder and that several reproductive milestones correlate with a change in migraine frequency or type, implicates sex hormones in the pathogenesis of migraine. Sex steroids differentially influence MA and MwA.

The pathogenesis of MM as of all changes in migraine related to hormones has not been fully characterized but appears to be related to estrogen withdrawal [19, 64]. The original study that reported the potential influence of estrogen withdrawal on headache was done over 30 years ago [65]. Women were selected based on their susceptibility to have MM; female migraineurs who were given estradiol had a delay in their migraine attacks until the level of estradiol dropped to pretreatment levels. A second study by the same author showed that treatment with progesterone resulted in a delay in bleeding and no change in the headache pattern [65]. These results served to postulate that the drop in estrogen was the cause for migraine in vulnerable women. Additional clinical evidence supports the role of estrogen withdrawal as a trigger for MwA. Several biological conditions associated with a fall in estrogens are repeatedly shown to be associated with a worsening of MwA: immediately before menstruation, during the pill-free period in women using COC or oral HRT, in hysterectomized women with bilateral oophorectomy, and delivery. Sudden estrogen withdrawal was associated with migraine also in women who received gonadotropin-releasing hormone as part of in vitro fertilization [66]. Continuous COCs use decreased headache dramatically [67]. On the opposite, high estrogen levels as occurring in the second and third trimester of pregnancy or their complete withdrawal such as in menopause, protect against MwA. Perimenopause, a time when the levels of circulating sex hormones fluctuate irregularly, is often associated with worsening or change in migraine patterns. At variance, conditions associated with high estrogen levels may lead to the development of MA. This occurs in women starting COCs, HRT, and during pregnancy. Reasons that may explain why some women are prone to migraine onset or worsening in relation to hormonal changes while others are not, are currently unclear. Differences may be related to the ability to metabolize estrogens or to polymorphism in genes encoding for sex hormones, their receptors, or metabolites of the hormonal pathways. As an example, a single nucleotide polymorphism in the estrogen receptor 1 gene G594A exon 8 [68] and the presence of a polymorphism of the progesterone receptor positively correlated with the incidence of migraine attacks [69].

Estrogens may interfere with cellular excitability or cerebral vessels. Ovarian steroids cross the blood–brain barrier by passive diffusion, with brain levels mirroring blood levels [70] and are also produced within the central nervous system [71]. Estrogen and progesterone can influence the pain-processing networks and the endothelium involved in the pathophysiology of migraine. Interrelationships between estrogens and brain neurotransmitters have been confirmed, including serotonin, norepinephrine, dopamine, and endorphins [42, 72]. In particular, estrogen has potent effects on the serotonergic system, increasing serotonergic tone. Prostaglandins have also been implicated in MM [73]. In particular, entry of prostaglandins into the systemic circulation can trigger throbbing headache, nausea, and vomiting [74]. Estrogen facilitates the glutaminergic system, potentially enhancing neural excitability. This effect is modulated by progesterone, which appears to activate GABAergic systems, suppressing neuronal reactivity [75]. Induction of cortical spreading depression (CSD) which is involved in the pathophysiology of migraine, depends on glutamatergic transmission [76, 77]. In addition, peak estrogen levels are associated with a significant decrease in serum Mg^++^ levels that could facilitate *N*-Methyl-D-Aspartate (NMDA) channel opening [78]. Changing central opioid tonus has been proposed as another mechanism that may induce migraine around the time of menstruation [79]. In contrast to women without MM, patients with MM exhibit poor response to LH after being injected with the opiate antagonist naloxone during the luteal phase of the menstrual cycle [80]. In an analysis of opioid tonus in women with MM, plasma β-endorphin and cortisol responses were impaired during the premenstrual period [72], indicating that premenstrual opioid hyposensitivity may contribute to the risk of MM. Estrogens may be involved in migraine pathophysiology also affecting the vasculature through stimulation of nitric oxide (NO) release [75, 81]. The estrogen receptor α increases NO synthase activity in the endothelium [82]. Women with a history of MM, with respect to women with migraine unrelated to the menstrual cycle or without migraine exhibit a heightened activation of the NO and l-arginine pathway and an increase in NO, especially during the luteal phase [83].

Estrogens may enhance susceptibility to MA by increasing cortical excitability. Studies of effects of estrogen on seizure threshold support this hypothesis. Estradiol enhances seizure susceptibility in females by decreasing the after-discharge threshold and facilitating kindling [84]. In patients with catamenial epilepsy, transcranial magnetic stimulation of motor cortex demonstrated a shorter cortical silent phase reflecting decreased inhibition during the luteal phase and during menstruation [85].

## Migraine, hormones and the risk of vascular diseases

### Migraine as a risk factor for vascular diseases

The higher-than-expected incidence of vascular disease (VD) reported in migraineurs suggests that migraine may, in some cases, be a dangerous condition rather than just a troublesome, but innocent, disorder [86–89] (Table 3). Available data support an increased risk of ischemic and hemorrhagic stroke, cardiac disease, retinal vasculopathy, and mortality in migraineurs suffering from MA [88, 89]. Several alternative conditions including thrombophilia, patent foramen ovale, arterial dissection, and autoimmunity have been advocated in the attempt to explain the high burden of VD in migraine but none of them fully explains the risk [88, 89]. This increased risk is not linked to any of the conventional vascular mechanisms underlying the above conditions, but rather to a specific systemic vascular vulnerability that is associated with migraine [90]. Whereas, there exists a huge body of evidence on the association between VD and migraine in women, data on migraine in men are scarce and lacking in detail, particularly as regards migraine type. Since no direct estimates of the risk in men versus women are available, it is impossible to establish whether the risk is higher in one of the two sexes. Bearing these limitations in mind, the available evidence suggests a definite increase in the risk of vascular events in the cerebral and cardiac districts in women suffering from MA while in men the same evidence is not definite. A recent meta-analysis showed an increased risk of ischemic stroke in women with any migraine versus women with no migraine, but not in men with any migraine versus no migraine [91]. According to incidence curves for ischemic stroke in the Women’s Health Study (WHS) and men in the Physicians’ Health Study (PHS), the association between migraine and ischemic stroke becomes more apparent over time in women, whereas in men it diminishes [92, 93]. This pattern suggests the involvement of different concurrent mechanisms according to gender. In both the WHS and the PHS, the presence of any migraine was associated with a greater risk of major vascular events, mostly due to an increase in myocardial infarction (not in ischemic stroke) [92, 93]. Women, but not men, were also found to be at increased risk of coronary revascularization, angina, and death from VD. In the American Migraine Prevalence and Prevention study, in both sexes stroke was more likely to occur in subjects with MA than in those with MwA. The same study reported an increased risk of vascular events in both men and women affected by any migraine, MA, and MwA [94]. Although, as mentioned, direct comparisons between men and women are lacking, the odds ratios were higher in men than in women, while confidence intervals were mostly overlapping. Data on mortality are conflicting. In the WHS, women affected by MA showed an increased risk of death from VD, while in the PHS, men suffering from any migraine did not present an increased risk of death from VD [92, 93]. Conversely, in the Reykjavik study, mortality from VD was marginally greater in men than in women affected by any migraine and by MwA [95]. A more recent meta-analysis assessed the relationship between migraine and mortality in ten cohort studies, in four of which MA and MwA were investigated separately [96]. The analyzed studies showed medium-to-high heterogeneity. The results indicated that the presence of any migraine did not alter the risk of all-cause, vascular, or coronary artery disease mortality. They also indicated that MA, but not MwA, increased the risk of VD and coronary artery disease mortality.

**Table 3:** Migraine and the risk of vascular disease

Ischemic stroke
Numerous studies demonstrating an association with migraine with aura^w1–18^
No definite association with migraine without aura
Association with migraine with aura confirmed by three meta-analyses^w19–21^
Hemorrhagic stroke
A single large study indicating an association with migraine with aura^w22^; other studies providing conflicting results^w23–26^
Cardiac events
Two large studies indicating an association with any migraine in men and women and with migraine with aura in women (data not available for men)^w3–4^; conflicting results provided by other available studies^w27–29^
No association with any migraine in meta-analysis of data^w30^; no analysis according to migraine type due to lack of data
Vascular death
A meta-analysis^w20^ and a large study^w31^ supporting an association with migraine with aura No association with any migraine according to meta-analysis of data^w32^
Other vascular diseases
Studies indicating a possible association with any migraine and retinal disease and peripheral artery disease^w33–39^

### Vascular effects of estrogens

The disparities in VD between premenopausal women and men of the same age suggest that endogenous sex hormones have a major vascular action and a protective effect from VD in women. Estrogens through receptor-operated mechanisms regulate peripheral arterial function, lipid metabolism, inflammation, oxidative stress, fibrinolysis, and thrombosis [97–99]. Estrogen receptors are expressed in myocardial cells, vascular smooth muscle cells, and endothelial cells in both humans and animals [99]. Antiatherogenic effects of estrogens are partially due to inhibition of vascular smooth muscle cells growth, proliferation, and contractility and may also affect endothelial cell regeneration and angiogenesis [100–103]. Several observations suggest that estrogens modulate endothelium-dependent relaxation by increasing vascular NO release. Basal vascular release of NO is generally higher in vessels derived from females than in those from males, and this difference is due to female sex hormones [104]. Both pregnancy and estradiol treatment increase endothelial NO synthase (eNOS) activity [105]; estradiol also increases eNOS activity and NO production in cultured endothelial cells [106, 107]. Moreover, estrogens reduce mitochondrial generation of reactive oxygen species [98]. In the absence of estrogen, endothelium-dependent release of NO is reduced, and the ability of estrogen to increase this response depends on the time-period an individual is without estrogen exposure [98]. However, estrogen signaling pathways are altered in older women, particularly in those with subclinical vascular disease, in a manner that converts vasoprotective effects to vasculotoxic effects. In fact, in premenopausal women, atherosclerotic coronary arteries express considerably less estrogen receptors than do normal arteries [108], suggesting that atherosclerosis is associated with diminished estrogen receptors expression, and that the antiatherogenic effects of estradiol are in part mediated through cardiovascular estrogen receptors [102, 108].

In cardiovascular tissues, as in others, estrogen can induce progesteron receptor (PR) expression [109]. Progestins inhibit estradiol-induced endothelium-mediated vascular relaxation [110], increase LDL and decrease HDL cholesterol levels [99], and high doses of progesterone also nullify the ability of estradiol to reduce intimal plaque size and cellular proliferation in a rabbit model of experimental atherosclerosis [111, 112]. Progesterone stimulates thrombospondin-1 expression by both endothelial cells and vascular smooth muscle cells, which potentially inhibits endothelial cell adhesion, migration, proliferation, and angiogenesis [113]. In contrast, micromolar concentrations of progesterone have been reported to induce endothelium-dependent relaxation of rabbit coronary artery [114] and to inhibit the induction of platelet calcium responses [115].

### Hormonal treatments and vascular diseases

While endogenous female hormones have a protective role with respect to VDs, exogenous hormones have, in most cases, neutral or deleterious effects. Many studies have suggested an increased risk of venous and arterial complications associated with COCs, primarily pills containing high doses of ethinylestradiol, raising concerns about their use. The risks of venous thromboembolism have been well established [116] while the risks of arterial diseases are more controversial. The risk of arterial disease is related to the dose of ethinylestradiol in the formulation. Evidences show that high-dose COCs (containing ≥ 50 μg of ethinylestradiol) are associated with an elevated risk of ischemic stroke while low-dose formulations (<50 μg of ethinylestradiol) are associated with a lower increase in the risk of stroke; data are not as clear regarding the risk of stroke associated with 20 μg versus 30 or 35 μg formulations [117]. Similarly, there are conflicting data regarding whether type of progesterone influences stroke risk in low-estrogen formulations. Second-generation progestogens (e.g., ethynodiol acetate, levonorgestrel, and norethisterone) are associated with a greater increase in the risk of myocardial infarction and ischemic stroke than third-generation progestogens (desogestrel, gestodene, norgestimate). For most women, COCs are a safe and highly effective method of contraception with added non-contraceptive health benefits. High-dose COCs, particularly those containing first-generation progestogens, are no longer recommended for routine use and low-dose formulations containing either second- or third-generation progestogens should be used, where possible. Users at a greater risk for venous or arterial vascular diseases include women over 35 years of age, heavy smokers, as women with high or abnormal blood lipids, with severe diabetes arterial damage, with consistently elevated blood pressure values, who are obese, or who suffer from migraine [55, 118]. Many studies have reported increased odds of stroke in migraineurs who use COCs [119–125]. According to a meta-analysis of available data, women with a history of migraine who use COCs are two to four times as likely to have an ischemic stroke as nonusers with history of migraine [126]. Reported odds ratios for ischemic stroke in migraineurs using combination contraception range from 2 to nearly 14, compared with nonusers (Table 4). A recent systematic review and meta-analysis of 9 studies found that the pooled relative risk of ischemic stroke in women aged less than 45 years with any migraine was 3.6, and the risk of ischemic stroke was further increased to 7.2 among women currently using COCs [91]. Coexistence of risk factors such as, COCs use and smoking has more than multiplicative effects on the odds ratios for ischemic stroke associated with migraine; the reported odds ratio of 34 for ischemic stroke among migraineurs women who use oral contraceptives and smoke is a matter of considerable concern [119]. At variance, high blood pressure showed a more linear relationship with the risk of ischemic stroke in migraineurs [119]. No studies had enough power to examine the risk of stroke by COC use and type of migraine (with or without aura) simultaneously. However, available evidences may suggest that COCs use may exacerbate the underlying vascular pathological condition of migraineurs.

**Table 4:** Risk of ischemic stroke in women by migraine status and COCs use

	Type of contraceptive	Risk of ischemic stroke OR; 95% CI			
		Women with migraine using COCs vs. women without migraine not using COCs	Women with migraine not using COCs vs. women without migraine not using COCs	Women with migraine using COCs vs. women with migraine not using COCs	Women without migraine using COCs vs. women without migraine not using COCs
Collaborative Group [120]^a^	High dose COCs			5.9; 2.9–12.2	4.9; 2.9–8.3
Tzourio [125]	Progestogen only High and low dose COCs	13.9; 5.5–35.1	3.7; 1.5–9.1		3.5; 1.5–8.3
Schwartz [124]	Low dose COCs			2.08; 1.19–3.65	0.88; 0.44–1.76
Chang [119]	High and low dose COCs	16.9; 2.72–106	2.27; 0.69–7.47		2.76; 1.01–7.55

*OR* odds ratio, *CI* confidence interval, *COCs* combined oral contraceptives

^a^Values represent relative risk and 95% CI

There have been many studies and clinical trials conducted in an attempt to address whether or not there is an increased incidence of VD among postmenopausal women on HRTs. The results of the Women Health Initiative study on HRTs and VD concluded that HRTs have no protective effect on the cardiovascular system. It has been suggested that HRTs may even promote the development of vascular events [127–130]. Transdermal estrogen has been postulated to be safer than oral estrogen with respect to stroke risk because it involves no exposure to first-pass liver metabolism and no increase in clotting factors and inflammatory markers when delivered across the skin [131]. There are no trial data directly comparing stroke risk with varying types and doses of estrogen as existing clinical trials were based on single regimens of HRT. Data from available observational studies are conflicting since some of them indicate a similar stroke risk between subjects using high and low doses of estrogen while other studies show a dose–response association [132–134]. Clinical trial results show that the use of either estrogen plus progestin or estrogen alone (taken orally) increases stroke risk in post-menopausal women [134–136].

## Prescription of hormonal treatments to migraineurs

Given the increased risk of VD in women with migraine and the demonstrated increased risk of stroke in women who use COCs, concern about their use in women with migraine has been raised and several guidelines report indications about prescription of COCs to migraineurs (Table 5) [137–140]. There are currently no studies that can be used to develop an evidence-based approach to the prescription of COCs to migraineurs. Physicians, when prescribing COCS, should take into account the type of migraine and the additional risk factors. Since MwA is not a definite risk factor for stroke, no particular restrictions are warranted in women suffering from this condition, especially in those with no comorbid risk factors. On the contrary, oral contraceptives should be discouraged in women suffering from MwA since they may lead to a further increase in the vascular risk. Their prescription should certainly be contraindicated in women with MA and other comorbid vascular risk factors or congenital or acquired thrombophilia. The additional risk factors for ischemic stroke in women with migraine using COCs are age >35 years, ischemic heart disease, or cardiac disease with embolic potential, diabetes mellitus, family history of arterial disease, hyperlipidemia, hypertension, obesity (body mass index >30), smoking, and systemic diseases associated with stroke, including sickle cell disease and connective tissue disorders [138]. As the risk of stroke also increases with age, COCs use is also considered inappropriate for women with any type of migraine over the age of 35 years; moreover, women who smoke should be advised to quit smoking before starting COCs. No specific tests need to be undertaken other than those routinely performed or indicated by the patient’s history or the presence of specific symptoms [138]. In some instances, starting COCs may be associated with a change or worsening in migraine. To date, the question of whether this change amplifies the risk of stroke in women has not been formally addressed but, as already suggested [138], new onset of migraine aura, new persisting headache, increased headache frequency or intensity, development of unusual aura symptoms, prolonged aura may necessitate further evaluation or cessation of COCs. The World Health Organization (WHO) considered progesterone-only contraceptives to be safer than COCs in subjects suffering from migraine [140], although migraine has not been considered when studying the safety, in terms of vascular risk, of those types of contraceptives. Intrauterine devices, particularly copper-bearing devices with respect to levonorgestrel-releasing devices are considered even safer than other possible hormonal contraceptive methods [140]. An awareness-raising strategy on prescription of COCs to migraineurs is needed, which should target gynecologists who are the specialists most likely to prescribe COCs. Indeed, gynecologists are often unskilled to diagnose migraine and unaware of the association between migraine and VD as of the implications of COCs use in migraineurs.

**Table 5:** Guidelines about prescription of combined oral contraceptives in migraineurs

International Headache Society Task Force on Combined Oral Contraceptives & Hormone Replacement Therapy [138]	World Health Organization [140]^a^	American College of Obstetricians and Gynecologists (2006)
There is no contraindication to the use of COCs in women with migraine in the absence of migraine aura or other risk factors. Women should be counseled and regularly assessed for the development of additional risk factors There is a potentially increased risk of ischemic stroke in women with migraine who are using COCs and have additional risk factors which cannot easily be controlled, including migraine with aura. One must individually assess and evaluate these risks. Combined oral contraceptive use may be contraindicated. Identify and evaluate risk factors (1) Identify and evaluate risk factors (2) Diagnose migraine type, particularly the presence of aura (3) Women with migraine who smoke should stop smoking before starting COCs (4) Other risk factors, such as hypertension and hyperlipidemia, should be treated (5) Consider non-ethinyloestradiol methods in women who are at increased risk of ischemic stroke, particularly those who have multiple risk factors. Some of these contraceptives are as or more effective in preventing pregnancy than COCs and include progestogen-only hormonal contraception. Observational studies suggest that progestogen-only hormonal contraceptive use is not associated with an increased risk of ischemic stroke, although quantifiable data are limited	Clarification: classification depends on accurate diagnosis of those severe headaches that are migrainous and those that are not. Any new headaches or marked changes in headaches should be evaluated. Classification is for women without any other risk factors for stroke. Risk of stroke increases with age, hypertension and smoking Evidence: among women with migraine, women who also had aura had a higher risk of stroke than those without aura. Among women with migraine, those who used COCs had a two to fourfold increased risk of stroke compared with women who did not use COCs (1) Non-migrainous headache (mild or severe) Initiation of COCs: a condition for which there is no restriction for the use of the contraceptive method ⇒Use method in any circumstances Continuation of COCs: a condition where the advantages of using the method generally outweigh the theoretical or proven risks ⇒Generally use the method (2) Migraine without aura and age <35 Initiation of COCs: a condition where the advantages of using the method generally outweigh the theoretical or proven risks ⇒Generally use the method Continuation of COCs: a condition where the theoretical or proven risks usually outweigh the advantages of using the method ⇒Use of method not usually recommended unless other more appropriate methods are not available or not acceptable (3) Migraine without aura and age ≥35 Initiation of COCs: a condition where the theoretical or proven risks usually outweigh the advantages of using the method ⇒Use of method not usually recommended unless other more appropriate methods are not available or not acceptable Continuation of COCs: a condition which represents an unacceptable health risk if the contraceptive method is used ⇒Method not to be used (4) Migraine with aura at any age Initiation and continuation of COCs: a condition which represents an unacceptable health risk if the contraceptive method is used ⇒Method not to be used	The use of COCs may be considered for women with migraine headaches if they do not have focal neurologic signs, do not smoke, are otherwise healthy, and are younger than 35 years. Although cerebrovascular events rarely occur among women with migraines who use combination oral contraceptives, the impact of a stroke is so devastating that clinicians should consider the use of progestin-only, intrauterine, or barrier contraceptives in this setting (Level B)

*COCs* combined oral contraceptives

^a^Recommendations refer to low-dose combined oral contraceptives <35 μg of ethinylestradiol

According to the International Headache Society Task Force on Combined Oral Contraceptives & Hormone Replacement Therapy [138] there is no evidence that migraine is a risk factor for ischemic stroke in women over 45 years of age. There are insufficient data to support an increased risk of ischemic stroke in women with any type of migraine who are using HRT. Consequently, the usual indications and contraindications for HRT should be applied. New onset headache should be carefully evaluated for secondary causes. If aura starts for the first time, transient ischemic attack should be excluded. The dose and route of delivery of estrogen replacement should be assessed to provide the lowest effective dose necessary to control menopause symptoms. If aura does not resolve, withdrawal of estrogen and non-hormonal strategies should be considered.

## Electronic supplementary material

Below is the link to the electronic supplementary material.

Supplementary material 1 (DOCX 21 kb)
