# Supplementary material for: Migraine in women: the role of hormones and their impact on vascular diseases
Source: J Headache Pain. 2012 Feb 26;13(3):177–89. doi: 10.1007/s10194-012-0424-y (PMC3311830; doi:10.1007/s10194-012-0424-y)
Supplement: Supplementary file 1 — Supplementary material 1 (DOCX 21 kb) [file 10194_2012_424_MOESM1_ESM.docx]

1. Henrich JB, Horwitz RI (1989) A controlled study of ischemic stroke risk in migraine patients. [J Clin Epidemiol](http://www.ncbi.nlm.nih.gov/pubmed/2760669) 42:773-780.
2. Merikangas KR, Fenton BT, Cheng SH, Stolar MJ, Risch N (1997) Association between migraine and stroke in a large-scale epidemiological study of the United States. Arch Neurol 54:362-368.
3. Kurth T, Gaziano JM, Cook NR, Logroscino G, Diener HC, Buring JE (2006) Migraine and risk of cardiovascular disease in women. JAMA 296:283-291.
4. Kurth T, Gaziano JM, Cook NR, Bubes V, Logroscino G, Diener HC, Buring JE (2007) Migraine and risk of cardiovascular disease in men. Arch Intern Med 167:795-801.
5. Lidegaard O, Kreiner S (2002) Contraceptives and cerebral thrombosis: a five-year national case-control study. Contraception 65:197-205.
6. Lidegaard O (1995) Oral contraceptives, pregnancy and the risk of cerebral thromboembolism: the influence of diabetes, hypertension, migraine and previous thrombotic disease. Br J Obstet Gynaecol 102:153-159.
7. MacClellan LR, Giles W, Cole J, Wozniak M, Stern B, Mitchell BD, Kittner SJ (2007) Probable migraine with visual aura and risk of ischemic stroke: the stroke prevention in young women study. Stroke 38:2438-2445.
8. Marini C, Carolei A, Roberts RS, Prencipe M, Gandolfo C, Inzitari D, Landi G, De Zanche L, Scoditti U, Fieschi C (1993) Focal cerebral ischemia in young adults: a collaborative case-control study. Neuroepidemiology 12:70-81.
9. Nightingale AL, Farmer RD (2004) Ischemic stroke in young women: a nested case-control study using the UK General Practice Research Database. Stroke 35:1574-1578.
10. Stang PE, Carson AP, Rose KM, Mo J, Ephross SA, Shahar E, Szklo M (2005) Headache, cerebrovascular symptoms and stroke: the Atherosclerosis Risk in Communities Study. Neurology 64:1573-1577.
11. Tzourio C, Tehindrazanarivelo A, Iglésias S, Alpérovitch A, Chedru F, d'Anglejan-Chatillon J, Bousser MG (1995) Case-control study of migraine and risk of ischaemic stroke in young women. BMJ 310:830-833.
12. Tzourio C, Iglesias S, Hubert JB, Visy JM, Alpérovitch A, Tehindrazanarivelo A, Biousse V, Woimant F, Bousser MG (1993) Migraine and risk of ischaemic stroke: a case-control study. BMJ 307:289-292.
13. Carolei A, Marini C, Nencini P, Gandolfo C, Motto C, Zanette E, Prencipe M, Fieschi C (1995) Prevalence and outcome of symptomatic carotid lesions in young adults. BMJ 310:1363-1366.
14. Carolei A, Marini C, De Matteis G (1996) History of migraine and risk of cerebral ischaemia in young adults. The Italian National Research Council Study Group on Stroke in the Young. Lancet 347:1503-1506.
15. Chang CL, Donaghy M, Poulter N. Migraine and stroke in young women: case-control study (1999) The World Health Organization Collaborative Study of Cardiovascular Disease and Steroid Hormone Contraception. BMJ 318:13-18.
16. Collaborative Group for the Study of Stroke in Young Women (1975) Oral contraceptives and stroke in young women: associated risk factors. JAMA 231:718-722.
17. Hall GC, Brown MM, Mo J, MacRae KD (2004) Triptans in migraine: the risks of stroke, cardiovascular disease, and death in practice. Neurology 62:563-568.
18. Becker C, Brobert GP, Almqvist PM, Johansson S, Jick SS, Meier CR (2007) Migraine and the risk of stroke, TIA, or death in the UK (CME). Headache 47:1374-1384.
19. Etminan M, Takkouche B, Isorna FC, Samii A (2005) [Risk of ischaemic stroke in people with migraine: systematic review and meta-analysis of observational studies.](http://www.ncbi.nlm.nih.gov/pubmed/15596418) BMJ 330:63.
20. Schürks M, Rist PM, Bigal ME, Buring JE, Lipton RB, Kurth T (2009) Migraine and cardiovascular disease: systematic review and meta-analysis. BMJ 339:b3914.
21. Spector JT, [Kahn SR](http://www.ncbi.nlm.nih.gov/pubmed?term=%22Kahn%20SR%22%5BAuthor%5D), [Jones MR](http://www.ncbi.nlm.nih.gov/pubmed?term=%22Jones%20MR%22%5BAuthor%5D), [Jayakumar M](http://www.ncbi.nlm.nih.gov/pubmed?term=%22Jayakumar%20M%22%5BAuthor%5D), [Dalal D](http://www.ncbi.nlm.nih.gov/pubmed?term=%22Dalal%20D%22%5BAuthor%5D), [Nazarian S](http://www.ncbi.nlm.nih.gov/pubmed?term=%22Nazarian%20S%22%5BAuthor%5D) (2010) Migraine headache and ischemic stroke risk: an updated meta-analysis. Am J Med 123: 612-624.
22. Kurth T, Kase CS, Schürks M, Tzourio C, Buring JE (2010) Migraine and risk of haemorrhagic stroke in women: prospective cohort study. BMJ 341:c3659.
23. Chang CL, Donaghy M, Poulter N (1999) Migraine and stroke in young women: case-control study. The World Health Organization Collaborative Study of Cardiovascular Disease and Steroid Hormone Contraception. BMJ 318:13-18.
24. Hall GC, Brown MM, Mo J, MacRae KD (2004) Triptans in migraine: the risks of stroke, cardiovascular disease, and death in practice. Neurology 62:563-568.
25. Bushnell CD, Jamison M, James AH (2009) Migraines during pregnancy linked to stroke and vascular diseases: US population based case-control study. BMJ 338:b664.
26. Carter KN, Anderson N, Jamrozik K, Hankey G, Anderson CS (2005) Australasian Co-operative Research on Subarachnoid Haemorrhage Study (ACROSS) Group. Migraine and risk of subarachnoid hemorrhage: a population-based case-control study. J Clin Neurosci 12:534-537.
27. [Cook NR](http://www.ncbi.nlm.nih.gov/pubmed?term=%22Cook%20NR%22%5BAuthor%5D), [Benseñor IM](http://www.ncbi.nlm.nih.gov/pubmed?term=%22Bense%C3%B1or%20IM%22%5BAuthor%5D), [Lotufo PA](http://www.ncbi.nlm.nih.gov/pubmed?term=%22Lotufo%20PA%22%5BAuthor%5D), [Lee IM](http://www.ncbi.nlm.nih.gov/pubmed?term=%22Lee%20IM%22%5BAuthor%5D), [Skerrett PJ](http://www.ncbi.nlm.nih.gov/pubmed?term=%22Skerrett%20PJ%22%5BAuthor%5D), [Chown MJ](http://www.ncbi.nlm.nih.gov/pubmed?term=%22Chown%20MJ%22%5BAuthor%5D), [Ajani UA](http://www.ncbi.nlm.nih.gov/pubmed?term=%22Ajani%20UA%22%5BAuthor%5D), [Manson JE](http://www.ncbi.nlm.nih.gov/pubmed?term=%22Manson%20JE%22%5BAuthor%5D), [Buring JE](http://www.ncbi.nlm.nih.gov/pubmed?term=%22Buring%20JE%22%5BAuthor%5D) (2002) Migraine and coronary heart disease in women and men. Headache 42:715-727.
28. [Ahmed B](http://www.ncbi.nlm.nih.gov/pubmed?term=%22Ahmed%20B%22%5BAuthor%5D), [Bairey Merz CN](http://www.ncbi.nlm.nih.gov/pubmed?term=%22Bairey%20Merz%20CN%22%5BAuthor%5D), [McClure C](http://www.ncbi.nlm.nih.gov/pubmed?term=%22McClure%20C%22%5BAuthor%5D), [Johnson BD](http://www.ncbi.nlm.nih.gov/pubmed?term=%22Johnson%20BD%22%5BAuthor%5D), [Reis SE](http://www.ncbi.nlm.nih.gov/pubmed?term=%22Reis%20SE%22%5BAuthor%5D), [Bittner V](http://www.ncbi.nlm.nih.gov/pubmed?term=%22Bittner%20V%22%5BAuthor%5D), [Pepine CJ](http://www.ncbi.nlm.nih.gov/pubmed?term=%22Pepine%20CJ%22%5BAuthor%5D), [Sharaf BL](http://www.ncbi.nlm.nih.gov/pubmed?term=%22Sharaf%20BL%22%5BAuthor%5D), [Sopko G](http://www.ncbi.nlm.nih.gov/pubmed?term=%22Sopko%20G%22%5BAuthor%5D), [Kelsey SF](http://www.ncbi.nlm.nih.gov/pubmed?term=%22Kelsey%20SF%22%5BAuthor%5D), [Shaw L](http://www.ncbi.nlm.nih.gov/pubmed?term=%22Shaw%20L%22%5BAuthor%5D); [WISE Study Group](http://www.ncbi.nlm.nih.gov/pubmed?term=%22WISE%20Study%20Group%22%5BCorporate%20Author%5D) (2006) Migraines, angiographic coronary artery disease and cardiovascular outcomes in women. [Am J Med.](http://www.ncbi.nlm.nih.gov/pubmed/16887413) 119:670-675.
29. [Logroscino G](http://www.ncbi.nlm.nih.gov/pubmed?term=%22Logroscino%20G%22%5BAuthor%5D), [Lipton RB](http://www.ncbi.nlm.nih.gov/pubmed?term=%22Lipton%20RB%22%5BAuthor%5D) (2004) Migraine is associated with chest symptoms but not cardiac events: a reassuring paradox. [Neurology](http://www.ncbi.nlm.nih.gov/pubmed/15623674) 63:2209-2210.
30. [Schürks M](http://www.ncbi.nlm.nih.gov/pubmed?term=%22Sch%C3%BCrks%20M%22%5BAuthor%5D), [Rist PM](http://www.ncbi.nlm.nih.gov/pubmed?term=%22Rist%20PM%22%5BAuthor%5D), [Bigal ME](http://www.ncbi.nlm.nih.gov/pubmed?term=%22Bigal%20ME%22%5BAuthor%5D), [Buring JE](http://www.ncbi.nlm.nih.gov/pubmed?term=%22Buring%20JE%22%5BAuthor%5D), [Lipton RB](http://www.ncbi.nlm.nih.gov/pubmed?term=%22Lipton%20RB%22%5BAuthor%5D), [Kurth T](http://www.ncbi.nlm.nih.gov/pubmed?term=%22Kurth%20T%22%5BAuthor%5D) (2009) Migraine and cardiovascular disease: systematic review and meta-analysis. [BMJ](http://www.ncbi.nlm.nih.gov/pubmed/19861375) 339:b3914.
31. Gudmundsson LS, Scher AI, Aspelund T, Eliasson JH, Johannsson M, Thorgeirsson G, Launer L, Gudnason V (2010) Migraine with aura and risk of cardiovascular and all cause mortality in men and women: prospective cohort study. BMJ 341:c3966.
32. [Schürks M](http://www.ncbi.nlm.nih.gov/pubmed?term=%22Sch%C3%BCrks%20M%22%5BAuthor%5D), [Rist PM](http://www.ncbi.nlm.nih.gov/pubmed?term=%22Rist%20PM%22%5BAuthor%5D), [Shapiro RE](http://www.ncbi.nlm.nih.gov/pubmed?term=%22Shapiro%20RE%22%5BAuthor%5D), [Kurth T](http://www.ncbi.nlm.nih.gov/pubmed?term=%22Kurth%20T%22%5BAuthor%5D) (2011) Migraine and mortality: a systematic review and meta-analysis. [Cephalalgia](http://www.ncbi.nlm.nih.gov/pubmed/21803936) 31:1301-1314.
33. Coppeto JR, Lessell S, Sciarra R, Bear L (1986) Vascular retinopathy in migraine. Neurology 36:267-270.
34. Newman NJ, Lessell S, Brandt EM (1989) Bilateral central retinal artery occlusions, disk drusen, and migraine. Am J Ophthalmol 107:236-240.
35. Beversdorf D, Stommel E, Allen C, Stevens R, Lessell S (1997) Recurrent branch retinal infarcts in association with migraine. Headache 37:396-399.
36. Glenn AM, Shaw PJ, Howe JW, Bates D (1992) Complicated migraine resulting in blindness due to bilateral retinal infarction. Br J Ophthalmol 76:189-190.
37. Liew G, Mitchell P, Wong TY, Wang JJ (2006) Retinal vascular caliber and migraine: the Blue Mountains Eye study. Headache 46:997-1004.
38. Rose KM, Wong TY, Carson AP, Couper DJ, Klein R, Sharrett AR (2007) Migraine and retinal microvascular abnormalities: the Atherosclerosis Risk in Communities study. Neurology 68:1694-1700.
39. [Jurno ME](http://www.ncbi.nlm.nih.gov/pubmed?term=%22Jurno%20ME%22%5BAuthor%5D), [Chevtchouk L](http://www.ncbi.nlm.nih.gov/pubmed?term=%22Chevtchouk%20L%22%5BAuthor%5D), [Nunes AA](http://www.ncbi.nlm.nih.gov/pubmed?term=%22Nunes%20AA%22%5BAuthor%5D), [de Rezende DF](http://www.ncbi.nlm.nih.gov/pubmed?term=%22de%20Rezende%20DF%22%5BAuthor%5D), [Jevoux Cda C](http://www.ncbi.nlm.nih.gov/pubmed?term=%22Jevoux%20Cda%20C%22%5BAuthor%5D), [de Souza JA](http://www.ncbi.nlm.nih.gov/pubmed?term=%22de%20Souza%20JA%22%5BAuthor%5D), [Moreira Filho PF](http://www.ncbi.nlm.nih.gov/pubmed?term=%22Moreira%20Filho%20PF%22%5BAuthor%5D) (2010) Ankle-brachial index, a screening for peripheral obstructive arterial disease, and migraine-a controlled study. [Headache](http://www.ncbi.nlm.nih.gov/pubmed?term=jurno%20and%202010) 50:626-630.
40. [Schwartz SM](http://www.ncbi.nlm.nih.gov/pubmed?term=%22Schwartz%20SM%22%5BAuthor%5D), [Petitti DB](http://www.ncbi.nlm.nih.gov/pubmed?term=%22Petitti%20DB%22%5BAuthor%5D), [Siscovick DS](http://www.ncbi.nlm.nih.gov/pubmed?term=%22Siscovick%20DS%22%5BAuthor%5D), [Longstreth WT Jr](http://www.ncbi.nlm.nih.gov/pubmed?term=%22Longstreth%20WT%20Jr%22%5BAuthor%5D), [Sidney S](http://www.ncbi.nlm.nih.gov/pubmed?term=%22Sidney%20S%22%5BAuthor%5D), [Raghunathan TE](http://www.ncbi.nlm.nih.gov/pubmed?term=%22Raghunathan%20TE%22%5BAuthor%5D), [Quesenberry CP Jr](http://www.ncbi.nlm.nih.gov/pubmed?term=%22Quesenberry%20CP%20Jr%22%5BAuthor%5D), [Kelaghan J](http://www.ncbi.nlm.nih.gov/pubmed?term=%22Kelaghan%20J%22%5BAuthor%5D) (1998) Stroke and use of low-dose oral contraceptives in young women: a pooled analysis of two US studies. [Stroke](http://www.ncbi.nlm.nih.gov/pubmed/9804634) 29:2277-2284.
